# Content and Quality of Mobile Apps for the Monitoring of Musculoskeletal or Neuropathic Pain in Australia: Systematic Evaluation

**DOI:** 10.2196/46881

**Published:** 2023-09-13

**Authors:** Joshua Simmich, Megan Heather Ross, Nicole Emma Andrews, Atiyeh Vaezipour, Trevor Glen Russell

**Affiliations:** 1 RECOVER Injury Research Centre, The University of Queensland, Brisbane, Australia; 2 STARS Education and Research Alliance, Surgical Treatment and Rehabilitation Service (STARS), University of Queensland and Metro North Health, Brisbane, Australia; 3 School of Health and Rehabilitation Sciences, The University of Queensland, Brisbane, Australia; 4 Tess Cramond Pain and Research Centre, Royal Brisbane and Women’s Hospital, Metro North Hospital and Health Service, Brisbane, Australia; 5 Occupational Therapy Department, Royal Brisbane and Women’s Hospital, Metro North Hospital and Health Service, Brisbane, Australia

**Keywords:** pain, monitoring, digital health, mobile application, digital health, mobile app, pain management, pain level, chronic pain, smartphone, musculoskeletal pain, neuropathic pain, remote

## Abstract

**Background:**

Mobile apps offer a potential mechanism for people with persistent pain to monitor pain levels conveniently within their own environment and for clinicians to remotely monitor their patients’ pain. However, the quality of currently available apps and the usefulness of included features from a clinical perspective are not known.

**Objective:**

The aim of this study was to examine the content and quality of currently available smartphone apps designed for monitoring the intensity or presence of musculoskeletal or neuropathic pain.

**Methods:**

A systematic search was performed in the Australian Apple and Google Play stores. Apps were included if they were designed to monitor the intensity or presence of musculoskeletal or neuropathic pain and were available in the English language within the Australian app stores. Data pertaining to the intended use of the app and clinical population were extracted by using a custom-designed data extraction form, and app quality was assessed by using the 23-item Mobile App Rating Scale.

**Results:**

Of the 2190 apps screened, 49 met the inclusion criteria. Apps were primarily designed for adult users (36/49, 73%) with nonspecific musculoskeletal or neuropathic pain conditions, arthritis, and joint pain. All apps monitored pain intensity, with almost half (23/49, 47%) also specifying pain location. Overall, the mean quality scores from the Mobile App Rating Scale ranged from 1.5 to 4.4 (out of 5.0). Between 20% (10/49) and 22% (11/49) of apps involved clinicians, consumers, or both in their development, and 20% (10/49) had published literature related to the development or use of the app in clinical scenarios. Although 71% (35/49) had data sharing features, only 5 apps enabled client-clinician communication through the app.

**Conclusions:**

The overall quality of mobile apps that are currently available for monitoring pain intensity is acceptable. Presently, mobile apps for remote pain monitoring lack functionality for clinicians to view data between consults. Both users and clinicians should be aware of the limitations of these apps and make informed choices in using or recommending apps that best suit the clinical need.

## Introduction

### Background

Persistent or chronic pain has been recognized as a global public health priority [1]. It is estimated that 20% of the adult population experience pain globally [2]. Persistent pain is considered a stand-alone disease [3,4], with musculoskeletal pain being by far the most prevalent pain condition [5,6]. Persistent pain is linked to changes in neural signaling and reorganization of the brain’s structure and function [7]. It is well established that persistent pain can have a devastating effect on individuals, interfering with relationships, mental health, and the ability to engage in meaningful and important activities [8-11]. The condition is a significant contributor to the opioid crisis [12] and incurs substantial costs to society through health system expenditures, decreased productivity, decreased quality of life, and the need for the provision of informal care [13,14].

Persistent pain management services often experience considerable health service strain, as evidenced in Australia, where high demand for such services has resulted in prolonged waiting times to access care [15]. This reality underscores the importance of innovation to improve service delivery, which could potentially be achieved through digital health technology [16-18]. Digital health technologies could empower patients to self-manage within their own environment, thereby decreasing the need for hospital or clinic visits. Digital health could also facilitate the remote monitoring of up-to-date personalized data to improve service delivery by assisting with patient triage, decreasing waiting times of those with urgent needs, and facilitating more timely treatment decisions [19-21]. By enhancing self-management, reducing barriers to accessing care, and addressing existing inefficiencies in pain management services, digital health innovations may lead to the more effective management of persistent pain.

Self-management refers to the “ability to monitor one’s condition and to effect the cognitive, behavioural and emotional responses necessary to maintain a satisfactory quality of life” [22]. Monitoring outcomes of behavior, such as pain intensity, is therefore a critical but underused component of pain self-management [23], as well as an established behavior change technique in its own right [24]. Self-monitoring could help people with persistent pain to become more aware of any patterns in their pain. Self-monitoring could also help individuals to identify behaviors or circumstances that may change their pain and help them to make changes in their behavior or lifestyle that may reduce their pain levels, thereby gaining an enhanced feeling of self-control [25]. In addition, monitoring has the potential to improve information exchange between people with pain and their clinicians. People with persistent pain can struggle to accurately recall pain intensity or fluctuations beyond the past several days [26], often leading to the overestimation of past pain intensity [27,28]. By regularly tracking their pain intensity, individuals can provide more accurate data to their clinicians, who would be better placed to provide feedback and support with pain management strategies. Additionally, receiving feedback from others, especially trusted others such as clinicians, is also a key behavior change technique for chronic disease management [24]. Overall, the monitoring of pain can be a valuable technique for people with persistent pain, helping them to better understand the nature of their pain and communicate this to clinicians.

Mobile health (mHealth) apps are being explored as potential tools for management and monitoring in people with persistent pain [29]. Although evidence suggests that mHealth apps may improve communication between health care professionals and patients [30], increase patients’ engagement with their health [31], and lead to health benefits [29,32,33], there remains a need for the systematic evaluation of the content and quality of available mHealth apps that focus primarily on pain monitoring. The Mobile App Rating Scale (MARS) is a widely used and reliable tool for assessing the quality of mobile phone apps [34] and has been used to appraise the quality of mHealth apps for low back pain [35-38], shoulder pain [39], and neck pain [40], as well as those for cancer [41] and arthritis [42,43]. However, these prior appraisals have largely focused on the use of apps for the broader concept of the self-management of pain, whereas the monitoring of pain intensity is only 1 component of self-management. A previous evaluation found that only half of commercially available pain management apps had a monitoring feature and that such apps instead more commonly gave instructions on new techniques (such as exercises or stretches), encouraged goal-setting, and provided education about the link between behaviors and pain [44]. To date, existing research has been limited to investigating the content, but not the quality, of general pain monitoring apps [45] or investigating both the content and the quality of apps for monitoring general pain and cancer pain [41]. However, musculoskeletal pain is by far the single largest category of persistent pain, with cancer-related pain constituting only a small minority of pain cases. Additionally, persistent musculoskeletal pain may overlap with neuropathic pain [46,47]. Therefore, there is a need for and a gap in the literature regarding the evaluation of apps for tracking and monitoring musculoskeletal or neuropathic pain symptoms.

### Aim

The aim of this study was to systematically review and appraise the content and quality of currently available mobile phone apps that were primarily designed for monitoring musculoskeletal or neuropathic pain intensity over time.

## Methods

### Search Strategy and Selection

The Apple App Store and the Google Play Store were searched in Australia on February 2, 2022. The keywords used for the search were based on the most common pain conditions in Australia and Canada [48,49]. The following keywords were used: *pain*, *arthritis*, *headache*, *migraine*, *post surgery*, *fracture*, and *fibromyalgia*. Search results and app details (eg, developer, cost, and version date and number) were downloaded to a spreadsheet, using Python-based App Store and Play Store scrapers developed by the Digital Methods Initiative [50,51]. Search results were first manually screened, by 2 reviewers (JS and AV), for apps that appeared in both stores and were then uploaded to Covidence (Veritas Health Innovation Ltd) for initial screening based on the names and descriptions of the apps.

Apps were included if they were available for public use and if monitoring musculoskeletal or neuropathic pain over time was a primary focus of the app. Apps were excluded if they were for monitoring reproductive pain or cancer pain. Although *migraine* and *headache* were initially added as search terms, the large volume of apps designed specifically for monitoring migraine and headache symptoms led to the decision to consider these separately; therefore, such apps were also excluded. Apps were additionally excluded if they were considered generic health monitoring apps (ie, apps for monitoring many health symptoms, without pain as the primary focus) or were no longer publicly available in Australia at the time of data extraction or evaluation.

Four reviewers (JS, MHR, AV, and NEA) independently performed the initial screening based on the inclusion criteria. Apps that met the selection criteria, according to 2 reviewers, were downloaded onto Apple or Android devices for a full review (Multimedia Appendix 1 provides device details). Disagreements were resolved through discussion or consultation with a third reviewer.

### Data Extraction

Apps that met the inclusion criteria were purchased (if applicable) and downloaded. One reviewer extracted general information about the apps by using a custom-made data extraction spreadsheet, and the second reviewer checked the veracity of this information. Extracted information included details of consumer and clinician involvement in app development, target populations, app features (eg, gamification, symptoms monitored, and monitoring frequency), pain tracking features (eg, intensity, location, and description), additional app features (eg, mood, exercise, and physical activity tracking), and data sharing features.

### Quality Appraisal

Each mobile app was independently rated by 2 reviewers from a panel of 4, ensuring that all reviewers participated in the process. Reviewers rated each app on the MARS, which consists of 23 items across categories, namely engagement, functionality, aesthetics, information quality, and subjective quality [34]. The MARS was scored on a 5-point scale (1: inadequate; 2: poor; 3: acceptable; 4: good; 5: excellent), as per standard instructions for use. Mean scores were calculated for the first 4 categories (engagement, functionality, aesthetics, and information quality), and an overall mean score for the MARS was obtained by averaging these 4 means. As recommended [34], all reviewers viewed the MARS training material. App quality ratings were piloted by having all 4 reviewers rate the same app initially. Subsequently, all reviewers met to discuss the ratings for this app to ensure that they had the same interpretation of the rating scale and process, before proceeding to rate the remaining apps.

Interrater reliability for the MARS was calculated by using the 1-way random effects intraclass correlation (ICC; ICC [1,1]), under the assumption that the 2 reviewers rating each app were randomly selected from the larger population of 4 reviewers [52]. A score of greater than 0.7 on the ICC was considered to indicate acceptable reliability between reviewers, while a score of greater than 0.8 indicated good reliability and a score of greater than 0.9 indicated excellent reliability.

## Results

### Overview of Apps

**Figure 1.**
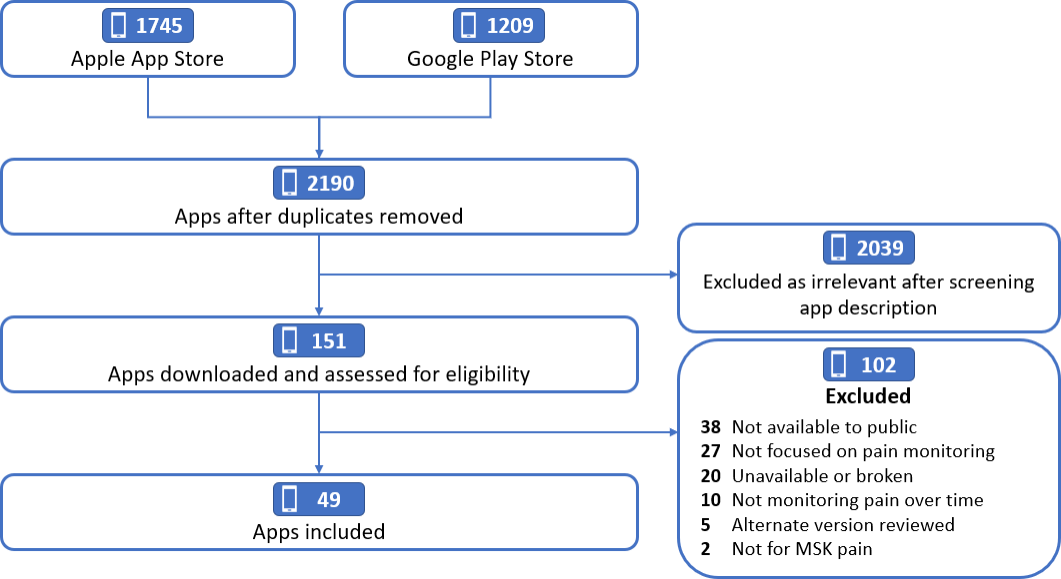
Flowchart of the app selection process. MSK: musculoskeletal.

Of the 2190 apps screened, 151 were downloaded for a full app review, and a total of 49 met the inclusion criteria and were screened by using the MARS. Mobile apps were primarily excluded for being designed for specific clinical studies or health facilities and not being designed primarily for pain monitoring (Figure 1). Details of the data extracted from the app stores are included in Multimedia Appendix 2, and the data extracted by reviewers for each mobile app are included in Multimedia Appendix 3.

In total, 35% (17/49) of included apps were available on both the Apple App Store and the Google Play Store, with 49% (24/49) only available in the App Store and 16% (8/49) only available in the Play Store. The majority of apps (35/49, 71%) were completely free to download and use, while an additional 10% (5/49) offered conditional free use but asked for payments to eliminate restrictions or advertisements. For those that required payment, the purchase cost ranged between Aus $1.49 (US $0.96) and Aus $10.99 (US $7.10), with some requiring an ongoing subscription or in-app purchases (Multimedia Appendix 2). Of the 49 included apps, 10 (20%) had published literature supporting their development, efficacy, or both; 10 (20%) were developed in consultation with clinicians; and 11 (22%) involved consumers in the design (Multimedia Appendix 3). Apps were primarily designed for use by adults (36/49, 73%), with only 3 (6%) designed for adolescents and 1 (2%) exclusively for children. Further, 5 (10%) used some form of gamification, primarily in the form of awarding points and achieving targets (for logging pain and activities).

### Pain Monitoring

The frequency at which users can enter pain ratings ranged from an unlimited number (ie, multiple entries per day) to the weekly logging of pain intensity, with 94% (46/49) of apps permitting at least daily recording. All apps recorded pain intensity (numeric rating scale: 24/49, 49%; visual analog scale: 19/49, 39%; Likert scale: 5/49, 10%; Wong-Baker Faces: 1/49, 2%) with a range of anchors and descriptors, including text, colors, and emojis (or faces). Around two-thirds (32/49, 65%) of apps graphically depicted change in pain symptoms over time with a chart or graph. Of the 49 apps, almost half (n=23, 47%) also recorded pain location, with 9 (18%) apps using a body chart to record pain location and the remaining 14 (29%) using text to describe the location of pain. Apps that recorded additional dimensions of pain were less frequent, with 15 (31%) recording pain type and quality and 10 (20%) recording duration and frequency. Reminders to enter data (ie, to monitor and track pain) were included in 18 (37%) of the apps, with the ability to customize the frequency of these reminders featured in 10 (20%) apps. Further details regarding the pain monitoring features of included apps are provided in Multimedia Appendix 3.

### Additional Features

Many apps included features that enabled users to monitor and track other symptoms and events, including medication (28/49, 57%), mood (25/49, 51%), and customizable or free-text notes (20/49, 41%). More detailed additional features are provided in Multimedia Appendix 4. Further, of the 49 apps, 20 (41%) included educational information or resources within the app or links to external resources for pain education, additional support, and further condition-specific information. All apps lacked the capacity to individualize management based on pain data entered by the patient; for example, they did not automatically adjust exercises or provide advice to manage a pain flare-up.

### Data Sharing

Of the 49 included apps, 35 (71%) had the ability to share data from the app (Multimedia Appendix 3). A wide range of file formats for exporting were used, with the most common being CSV and PDF formats (either as a file for the device or as an attachment in an email). Further, 5 apps had data sharing functionalities within the app platform itself, requiring clinicians to be a user of the app to receive shared data. No apps offered real-time data sharing with clinicians. Apps primarily shared raw data (34/49, 70%) and graphs (32/49, 65%), with fewer apps providing a summary of the data (18/49, 37%). Of the included apps, 32 had a privacy policy listed on the store or within the app, whereas 17 apps did not.

### MARS Ratings

The ICC (1,1) for agreement on the MARS ratings between reviewers was 0.72 (95% CI 0.61-0.81), indicating good reliability, but the uncertainty ranged from less than acceptable (<0.7) to better than good (>0.8). The median overall MARS score for included mobile apps was 3.1 (range 1.5-4.4). Almost 60% (29/49, 59%) of apps scored ≥3.0 (acceptable) overall; the highest mean score was for the functionality (mean 3.5, SD 0.66) domain (Multimedia Appendix 5). Included apps, on average, had the lowest scores for the engagement domain, with a mean of 2.5 (SD 0.63), which is below *acceptable* on the MARS.

## Discussion

### Principal Results

This study systematically reviewed the content and appraised the quality of currently available mobile phone apps that were primarily designed for monitoring musculoskeletal or neuropathic pain intensity over time. In an era where digital health solutions are increasingly prevalent, providing a snapshot of the landscape of pain monitoring apps is critical. These findings have important implications for the potential use of these apps in clinical practice from the perspectives of both clinicians and people with persistent pain.

In conducting this evaluation, we identified a range of apps that had different methods for highlighting painful body parts and monitoring pain over time, ranging from basic methods to sophisticated methods. In accordance with existing literature on general pain monitoring apps [45] and pain management apps [53], only 31% (15/49) of the included apps allowed users to rate or describe other dimensions of their pain, such as its quality. It is likely that unidimensional ratings of pain intensity are used for their simplicity for users and app developers, but it is critical to consider that pain is an inherently multidimensional experience. Perhaps underscoring the increasing attention to tracking other dimensions of the pain experience, medication use and mood were monitored in 57% (28/49) and 51% (25/49) of included apps, respectively, which are higher than the 39% and 31% reported in prior research [45]. Additionally, 65% (32/49) of the apps in our study used graphs or charts to visualize pain intensity, closely matching the 61% of apps that were noted to have data visualizations in a prior study of apps that tracked general or cancer pain [41]. Given how useful graphical representations are in summarizing an overall pattern in pain presentation, it is surprising that more apps have not adopted a graphical display.

We identified several limitations of the apps reviewed in this study. Of significant concern, only 20% (10/49) to 22% (11/49) of the apps included in this study had involved either clinicians or people with persistent pain in their design process. This aligns with prior findings indicating that only 31% of general pain monitoring apps consulted health care practitioners in development, and only 5.6% involved patients [45]. Though the apparent increase in the involvement of people with persistent pain is promising, as these individuals are ultimately the end users of these apps, the relatively low involvement of these key stakeholders is concerning and suggests that a greater focus is required for user-centered design and co-design. Moreover, we discovered that 4 out of every 5 apps (39/49, 80%) assessed in this study did not provide any publicly available literature outlining their development process or reporting any evaluation of validity, user perspectives, efficacy, or usage. Although this may not be entirely unexpected, considering the considerable costs and time investments necessary to undertake such research, it hampers the ability of consumers and health care professionals to make well-informed decisions when selecting or endorsing such apps. Finally, none of the included apps allowed clinicians to tailor any management components to the individual, despite there being some evidence that tailored technology interventions [54] and individualized pain management interventions [55,56] may be more effective and are valued by patients [57].

The MARS ratings for the included apps described the overall quality and quality indicators of the apps, including engagement, functionality, aesthetics, and information quality [34]. Scores for apps were highest for the functionality subscales and lowest for the engagement subscales. This pattern in MARS ratings has been consistently observed across several prior studies that appraised apps for the self-management of pain conditions [35,36,38,44,58]. Likewise, in apps for monitoring general or cancer pain, functionality was the highest-scoring domain, and engagement was the second-lowest–scoring MARS domain after information quality [41]. Therefore, despite the apps functioning well, the lack of features for promoting engagement may result in the inconsistent or discontinued use of the app for remote pain monitoring and, in turn, an incomplete picture of the pain presentation. Only 10% (5/49) of the apps included in this study had any gamification features, and in a prior investigation, gamification was not noted in any apps for the self-management of back pain [36]. Future apps and further updates to existing apps should consider embedding features and techniques that increase engagement (ie, gamification) to promote the desired frequency of monitoring.

An emergent finding of our review was that most apps (44/49, 90%) lacked a method for direct data sharing between users and clinicians (eg, facilitating live access via a dashboard or exporting data in a standard format that can be imported to electronic medical records). Instead, the apps usually saved a data file or graph (often to be printed or attached to an email). Considering that clinicians are concerned about implementing apps in a clinical context due to workflow disruptions and time burden [59,60], this has the potential to place significant burdens on clinicians who may be unable to deal with the reports generated by their patients, especially due to the potential for varied formats from different apps. The potential burden on clinicians could be further exacerbated by apps that export data in file formats that are not designed for human readability (such as JSON and CSV), which require the clinician to have the time and skills for converting these data to a format that makes them readable to a human and clinically meaningful. Data need to be presented in a format that is useful, usable, and interpretable for clinicians and users, such as a familiar data visualization format [61,62]. Access to a live dashboard or the sharing of standardized data in usable and clinician-friendly formats is recommended for future pain monitoring apps, as well as ensuring that robust security measures are put in place to protect sensitive health data and adhere to relevant data protection laws.

### Implications for Clinical Practice

Health professionals may opt to use a pain monitoring app for clients with varying outcomes in mind and for a variety of reasons. The appropriateness of using pain monitoring apps must be determined based on the client’s clinical presentation. For example, clients demonstrating high levels of pain catastrophizing may not be suitable candidates for ongoing pain monitoring, especially not without supervision and reassurance from clinicians, as pain monitoring may lead to increased focus on and worry about pain symptoms [63]. On the other hand, clients who are “overactive” and experience pain flare-ups may be able to use self-monitoring to better pace themselves [57,64]. Similarly, for clients who avoid activity due to the fear of provoking pain, the regular monitoring of pain may lead to improved confidence by correcting exaggerated predictions of pain provocation [65]. In this latter case especially, a mobile app that tracks activity, as well as pain monitoring, may be necessary to demonstrate that activity does not always lead to an increase in pain [63,65] and that safe and acceptable levels of pain can occur during exercise without causing harm, which are both important understandings to facilitate participation in rehabilitation (eg, tendinopathy rehabilitation [66]). Finally, clients who are making progress may be motivated by visualizing progress in easily comprehensible data outputs, such as graphs, which can facilitate focus on past successes—a known behavior change technique [24].

When pain monitoring apps are deemed appropriate, the selection of a specific app can be facilitated by the findings of this study. Clinical decisions regarding which app would be the most suitable for individual clients can be based on various factors, including the method of pain identification used within the app (such as the selection of broad body areas vs the ability to shade on a body chart), the requirements for monitoring additional dimensions of pain symptoms (such as neuropathic pain, tingling, or numbness), the availability of additional tracking features (such as those for exercises, physical activity, or mood), and the need for clinicians to access the data (such as real-time monitoring for high-level athletes vs weekly check-ins for an outpatient clinic setting). Given the tendency for clinicians to recommend mobile apps that patients are already using and are favorable toward [67], it could also be beneficial for patients’ familiarity and preferences for specific apps to be routinely considered to potentially enhance engagement.

### Implications for Future Research

This study revealed that a limited number of the evaluated apps were clearly grounded in evidence or research. This highlights the need for future research into the development of pain monitoring apps that are based on established clinical guidelines [68,69] and developed in consultation with consumers and clinicians. Rigorous randomized controlled trials remain the gold standard for assessing the effectiveness of mHealth apps but can be challenging to conduct with sufficient durations and sample sizes. Although the UK National Institute for Health and Care Excellence Evidence Standards Framework for Digital Health Technologies recommends formal trials for apps that aim to treat or diagnose health conditions [70], for health monitoring apps, the focus is on evidence of successful pilot tests within the health and care system that show relevance to current service provision or best practice. In addition to empirical evidence, app developers and researchers can reference frameworks, such as the Assessment Framework for mHealth Apps published by the Australian Digital Health Agency [71], to ensure that the app is deemed safe, trustworthy, useful, usable, and likely to be effective. Further research is also needed to investigate the real-world use and uptake of pain monitoring apps by individuals with persistent pain, as this information can inform the design and implementation of future apps to better meet the needs of users. Additionally, future research could also explore how apps can be tailored to specific populations, such as older adults, children, or individuals with specific pain conditions.

### Limitations

There are several limitations to this study that should be considered when interpreting the results. First, the search was conducted over 1 year prior to the date of publication. Although the results presented nonetheless provide a valuable snapshot of the app landscape at the time, the rapidly evolving nature of this field must be considered. It is likely that this study may include apps that have since been discontinued or substantially updated, and newly published relevant apps would be missing from our review. This omission could potentially limit the future applicability of our findings to clinicians and people with persistent pain. Second, our search was limited to the Australian Apple and Google Play stores and was conducted in English, which means that apps that are only available in other countries or languages were not included in this review. Third, we excluded apps that required users to log into a patient portal or medical practice website because we were unable to access these apps. It is possible that these apps may provide real-time access to data and improved management options for clinicians, as well as potentially better privacy protections. However, the aim of this study was to evaluate publicly available apps for monitoring pain; therefore, these apps were outside the scope of our review. Finally, agreement on the quality assessment using the MARS was lower than that in prior studies. This may be due, in part, to the fact that prior studies used the same reviewers for all apps, whereas in this study, each app was rated by just 2 of the 4 reviewers, which may have introduced an additional source of variability.

### Conclusions

This study reviewed mobile phone apps designed for monitoring pain intensity over time and found that while many apps with various features existed, they lacked the capacity for real-time data sharing with clinicians and were rated poorly for engagement. Many of the apps lacked any supporting research publications. This study suggests that future apps should focus on increasing engagement and providing data in a usable format for clinicians. The appropriateness of using pain monitoring apps should be determined based on the patient’s clinical presentation, as well as client preferences.

## Supplementary material

10.2196/46881 Multimedia Appendix 1 Hardware and system software used by app reviewers.

10.2196/46881 Multimedia Appendix 2 Details of data extracted from the app stores.

10.2196/46881 Multimedia Appendix 3 Data extracted by reviewing each app.

10.2196/46881 Multimedia Appendix 4 Additional features present in each included app.

10.2196/46881 Multimedia Appendix 5 Mobile App Rating Scale scores (average of 2 reviewers) for each included app.

## Abbreviations

ICC: intraclass correlation
MARS: Mobile App Rating Scale
mHealth: mobile health
