# Supplementary material for: Content and Quality of Mobile Apps for the Monitoring of Musculoskeletal or Neuropathic Pain in Australia: Systematic Evaluation
Source: JMIR Mhealth Uhealth. 2023 Sep 13;11:e46881. doi: 10.2196/46881 (PMC10510453; doi:10.2196/46881)
Supplement: Multimedia Appendix 1 [file mhealth-v11-e46881-s001.docx]

Multimedia Appendix 1: Hardware and system software used by app reviewers

| **Reviewer Initials** | **iOS Device (and OS version)** | **Android Device (and Android Version)** |
| --- | --- | --- |
| MR | iPhone 12 (iOS 15.5) | Not applicable |
| NA | iPad 5th Gen (iOS 12.4) | Samsung Galaxy Z Flip3 (Android version 13) |
| AV | iPhone 11 (iOS 15.6) | Samsung Galaxy Tab A 10.1 (Android 11) |
| JS | iPad 5th Gen (iOS 14.8.1) | LG Q7 (Android 9) |
